# Assessment of Patient's Satisfaction and Associated Factors regarding Postoperative Pain Management at the University of Gondar Compressive Specialized Hospital, Northwest Ethiopia

**DOI:** 10.1155/2020/8834807

**Published:** 2020-11-12

**Authors:** Yosef Belay Bizuneh, Girmay Fitiwi Lema, Demeke Yilkal Fentie, Yophtahe Woldegerima Berhe, Henos Enyew Ashagrie

**Affiliations:** Department of Anesthesia, College of Medicine and Health Sciences, University of Gondar, Gondar, Ethiopia

## Abstract

**Objective:**

We aimed to assess the level of patient's satisfaction and associated factors regarding postoperative pain management.

**Methods:**

An institution-based cross-sectional study was conducted from April to May 2018 at the University of Gondar, and comprehensive specialized hospital data were collected through semistructured questionnaire and chart review. Level of satisfaction was measured using five-point Likert scale. Statistical analysis was done using SPSS software version 23. Both bivariable and multivariable logistic regression analyses were done. Variables of *P* value ≤0.2 in the bivariable analysis were a candidate for multivariable logistic regression. A *P* value ≤0.05 was considered as significantly associated with patient's level of satisfaction at 95% CI.

**Results:**

A total of 418 patients were included in this study with a response rate of 98.58%. The overall proportion of patients who were satisfied with pain management services was 72.2% (95% CI: 67.7–76.6). ASA1 (AOR = 3.55: 95% CI = 1.20–10.55) and ASA2 patients (AOR = 3.72: 95% CI = 1.04–13.28), absence of postoperative pain (AOR = 1.86: 95% CI = 1.02–3.39), peripheral nerve block done (AOR = 9.14: 95% CI = 3.93 20.86), received analgesic before request (AOR = 6.90: 95% CI = 3.72–12.83), and received systemic analgesics (AOR = 6.10: 95% CI = 1.17–33.91) were significantly associated with the level of satisfaction.

**Conclusion:**

The level of patient satisfaction with postoperative pain management was considerably low. Hence, it is vital to implement time-interval pain assessment method during the first 24 hours of postoperative period and treat accordingly based on the WHO pain ladder. Moreover, we suggested that all patients who underwent major surgery should receive peripheral nerve block as part of multimodal analgesia to decrease the incidence and severity of post op pain.

## 1. Background

Globally, postoperative pain is one of the major concerns in surgical patients and many studies have been done through different methods to assess patient satisfaction with postoperative pain management and associated factors [1–3].

Satisfaction is a general psychological condition that results from emotional surrounding expectations coupled with the prior feeling of consumers toward the consumption experience [4]. Patient's satisfaction in pain management is one of the variables that affect the outcomes of health care services, but it could be difficult to achieve by a single specific intervention [5, 6]. Previously, traditional lack of complications and vital sign was used as a measurement of clinical success. However, recently, patient-reported outcome measures or patient satisfaction are the most relevant criteria of clinical success [7].

Studies suggested that pain management can be affected by multiple factors such as gender, age, preoperative expectations, information given prior to surgery, ASA status, preoperative pain medication, type of anesthesia, type and duration of surgery, communication of staffs with patient, and experience of pain relief [8–11]. Patients' level of satisfaction could be increased via providing preoperative information related to postoperative pain, preoperative education, and nonpharmacological treatment [12, 13].

Untreated postoperative pain may have an effect in the clinical and psychological status of the patients. In addition, it creates a burden to health institutions by increasing costs and prolonging hospital stay. In addition, untreated acute postoperative pain may change to chronic pain with sequelae of decreased quality of life with different clinical sequels [14–18].

Despite different methods of postoperative pain control have been provided to surgical patients, there had been lack of evidence that examined patients' satisfaction with the quality of postoperative pain management in the study area. Therefore, we aimed to assess the level of patient's satisfaction and identifying factor that could affect postoperative pain management.

## 2. Methods

### 2.1. Study Design, Area, and Period

Institution-based cross-sectional study was conducted from April to May 2018 at the University of Gondar Comprehensive Specialized Hospital, northwest Ethiopia. The University of Gondar Comprehensive Specialized Hospital is a referral and teaching hospital which is found in Gondar town, about 738 km northwest from Addis Ababa, capital of Ethiopia, and 230 km from Ethiopia–Sudan boarder. The hospital is estimated to serve over 5 million people around the area and according to the annual report of the hospital, more than six thousand patients were operated on per annum.

### 2.2. Sample Size Determination and Sampling Procedure

Sample size was calculated using a single population proportion formula. In a previous study done in Jimma University Specialized Hospital, it was shown that satisfaction rate was 50%, 5% of accepted difference, and CI of 95%:

(1) $$\begin{array}{c} n=\frac{{\left(\mathrm{Ζ\alpha}/2\right)}^{2}p\left(1-p\right)}{W^{2}}. \end{array}$$

Assumptions **n** = is the required sample size, **Z** = critical value for normal distribution at 95% confidence level (1.96), **W** = 0.05 (5% margin of error), ***α*** = the level of significance = best estimate of the population proportion, and 10% of nonresponse rate was added. Therefore, based on the abovementioned assumptions, 424 participants were enrolled in this study. All consecutive surgical patients were included until the calculated sample size was reached.

### 2.3. Data Collection Procedure

Data were collected through patients' chart review, interview, and five-point Likert scale. Questionnaires including five-point Likert scale were translated to local language (Amharic language) and pretest was done on 43 patients (10% of the estimated sample size) and amendment was done before the actual data collection.

Data was collected at 24 hours after operation. Two junior anesthetists were involved in data collection after receiving training and supervised by the principal investigator. The level of patient's satisfaction was measured using five-point Likert scale ((1 = very dissatisfied, 2 = dissatisfied, 3 = neutral, 4 = satisfied, and 5 = very satisfied) and checklist. The reliability coefficient (Cronbach *s*, *s*^−^alpha) test of this instrument was 0.97. The questionnaire was modified from the American Pain Society satisfaction survey, American Pain Society patient-outcomes questionnaires–modified [18–20], and the Department of Anesthesiology and Intensive Care, Helsinki University Hospital [11].

### 2.4. Study Variables

#### 2.4.1. Dependent Variables

Level of patient's satisfaction expressed through 5-point Likert scale. Patients' level of satisfaction with postoperative pain management was based on the demarcation threshold formula [total highest score total − lowest score]/2] + (total lowest score) [21–25]. Patient who scored less than 79.5 points out of 130 was considered as dissatisfied whereas 79.5 and above was considered as satisfied.

#### 2.4.2. Independent Variables

*(1) Sociodemographic Factors*. Sex, age, BMI, and level of education.

*(2) Preoperative Factors*. Previous surgical history, preoperative pain, treatment and previous side effects of pain medication, preoperative information of postoperative pain management, and disease status.

*(3) Surgical Related Factors*. Body site of surgery, type of surgery, type of anesthesia and analgesia, duration of surgery, surgeon, and intra- and postoperative pain.

### 2.5. Data Processing and Analysis

Data clean-up and cross-checking were done before analysis. The coded data were entered to Epi Info software version 7 and exported to SPSS version 23. Bivariate and multivariate logistic regression analyses were used to control the possible confounding factors and to identify factors associated with patient's satisfaction. The cut-off point for statistical significance was <0.2 and 0.05 for bivariate and multivariate logistic regression analyses, respectively. The relationship of nominal data with satisfaction was analyzed by using cross tabulations. Categorical data were presented as numbers and frequencies (percentages). Patient's satisfaction through five-point Likert scale was dichotomized in to satisfied and dissatisfied groups based on demarcation threshold formula.

## 3. Results

### 3.1. Sociodemographic and Clinical Characteristics of the Study Participants

A total of 418 patients with a response rate of 98.58% were enrolled in this study. Six patients were excluded from analysis for incomplete data. Two-thirds of the study subjects, 279 (66.7%), were females. Most of the respondents, 311 (74.4%), were in the age group of 18–39 years and the mean age ± SD was 33.5 ± 13.2 years and the mean ± SD of BMI was 22.2 + 3.0 kg/m^2^. The majority of the study participants, 64.4%, 87.1%, and 55%, underwent abdominal procedure, major surgery, and emergency surgery, respectively. More than fifty percent (54.1%), 45.9%, of patients were operated on under general anesthesia and 54.1% were operated on under spinal anesthesia (Table 1).

### 3.2. Level of Patient's Satisfaction in Postoperative Pain Management with Subscales and Overall

**Figure 1:**
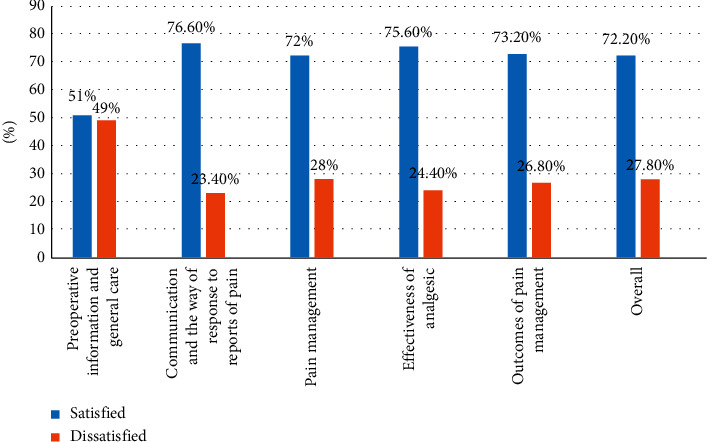
Level of patients' satisfaction with subscales and overall in postoperative pain management at the University of Gondar comprehensive specialized hospital, northwest Ethiopia, 2018 (*N* = 418).

The overall of patients who were satisfied with pain management service was 72.2% (95% CI: 67.7–76.6). The highest satisfaction score was with communication and the way of response to reports of pain (76.6%) whereas the lowest score was with preoperative information and general care (Figure 1).

### 3.3. Factors Associated with Patient's Satisfaction with Postoperative Pain Management

Multivariate analysis showed that ASA status, postoperative pain, nerve block, receive analgesics, and analgesics before request were the significant factors associated with patient satisfaction in postoperative pain management (Table 2).

## 4. Discussion

Previous studies showed that the level of patient satisfaction was not associated with postoperative pain severity, since the majority of the patients were considered satisfied, even though they had moderate-to-severe pain. In contrary, other studies showed that satisfaction was associated with postoperative pain management [26–29].

In the current study, the overall proportion of patients who were satisfied with pain management services was 72.2% (95% CI: 67.7–76.6). This finding was low compared with other studies [3, 8, 9, 11, 14, 26, 30–33]. This could be due to the good caring attitude of health care professional, high rate of preoperative pain education, presence of good communication, and providing frequent education on pain-related issues for the ward nurses, especially focusing on the frequent measurement of pain assessment [11, 34, 35]. In addition, nonpharmacological pain management methods were implemented but not in the study area [10]. However, our finding was high compared with the recent study of Jimma (Ethiopia), which showed that the overall proportion of patients satisfaction was 50% [36]. This discrepancy could be because the peripheral nerve block was a common method of pain management in the study area while this method was not mentioned in that study.

In the current study, we found that disease status has an association with the level of satisfaction, ASA1 patients were 3.5 times more likely to be satisfied compared with ASA3 and ASA4 patients (AOR = 3.55 (1.20–10.55)) and ASA2 patients were 3.7 times more likely to be satisfied compared with ASA3 and ASA4 patients (AOR = 3.72, 95% CI = 1.04–13.28). A study conducted in Australian patients showed that ASA3 and above were associated with patients' dissatisfaction in postoperative pain management survival in univariate odds ratio analysis. However, after adjustment, ASA status was no longer in associated with patients dissatisfaction [33].

Patients who received a postoperative regional analgesic technique generally had lower pain scores and a higher level of satisfaction [6, 37–41]. In the present study, 95.7% of patients were satisfied with postoperative nerve block, which was 9 times more likely to be satisfied compared with patients without nerve block (AOR = 9.14, 95% CI = 3.93–20.86). This showed that our study had similar finding with other studies [6, 37, 38, 40]. This is because of nerve block has superior postoperative analgesia, which may result in higher levels of patient's satisfaction [38].

From 418 patients, 15 (3.6%) had not received analgesics; among them, only 5 (33.3%) patients were satisfied whereas 10 (66.6%) patients were dissatisfied. When compared with patients who had received analgesic versus patients who had not received analgesics, patients who had received analgesic were 6 times more likely to be satisfied (AOR = 6.1: 95% CI = 1.17–33.91). Furthermore, in the other studies, it was shown that patients who had received analgesics postoperatively were highly satisfied compared with nontreated patients [20, 42].

In the current study, 149 (35.6%) patients had pain immediately after the operation. Those patients were 1.8 times less satisfied when compared with those who did not have pain immediately after the operation (AOR = 1.86: 95% CI = 1.02–3.39). Several studies conclude that patients' satisfaction with postoperative pain management was associated with the patients' actual pain experience [9, 19, 27, 34, 43], and our finding was in accordance with the abovementioned literatures.

Another factor associated with patient satisfaction in postoperative pain management was using analgesic before request. Patients who received analgesics before request were 6.9 times more likely to be satisfied compared with those patients who had not received analgesics before request or totally did not receive analgesic (AOR = 6.90: 95% CI = 3.72–12.83). This finding is also in line with the other study and could be the association between pain management and patient's satisfaction [44].

## 5. Conclusions

The overall level of patient's satisfaction with postoperative pain management was considerably low as compared with other studies. ASA status, presence of pain immediately after operation, peripheral nerve block, administration of systemic analgesics, and analgesic before request were significant determinant factors for patients' level of satisfaction. Hence, it is vital to implement time-interval pain assessment method during the first 24 hours of postoperative period and treat accordingly based on the WHO pain ladder. Moreover, we suggested that all patients who underwent major surgery should receive peripheral nerve block as part of multimodal analgesia to decrease the incidence and severity of post op pain.

### 5.1. Limitation of the Study

Dichotomized Likert data leads to loss of information and the space between each choice cannot possibly be equidistant. The study did not include patients discharged before 24 hours, and after 24 hours, the level of satisfaction was not assessed.

## Figures and Tables

**Table 1:** Sociodemographic and clinical characteristics of patients underwent surgery under anesthesia at the University of Gondar comprehensive specialized hospital, northwest Ethiopia, 2018 (*N* = 418).

Variables		Frequency (*n*)	Percentages (%)
Gender	Male	139	33.3
	Female	279	66.7
Age (years)	18–39	311	74.4
	40–55	73	17.5
	>55	34	8.1
BMI	Under weight	29	6.9
	Normal	325	77.8
	Over weight	53	12.7
	Obese	11	2.6
Education	Unable to read and write	216	51.7
	Able to read and write	8	1.9
	Primary school	43	10.3
	High school	83	19.9
	College/university	68	16.3
ASA status	ASA1	311	74.4
	ASA2	69	16.5
	ASA3 and ASA4	38	9.1
Body site of surgery	Limbs	47	11.2
	Head and neck	52	12.4
	Thoracic	8	1.2
	Abdomen	269	64.4
	Spine	4	1.00
	Genitourinary	38	9.1
Types of surgery	Elective	188	45
	Emergency	230	55
	Major	364	87.1
	Minor	54	12.9
Type of anesthesia	GA	192	45.9
	SA	226	54.1

Data were expressed in number and percentage. GA: general anesthesia. SA: spinal anesthesia.

**Table 2:** As assessed 24 h after surgery, bivariate and multivariate logistic regression analyses results, patients experiences in perioperative time for satisfaction of postoperative pain management, assessed at 24 hours following surgery in the University of Gondar compressive specialized hospital, northwest Ethiopia, 2018 (*N* = 418).

Variables		Level of satisfaction		Odds ratio with 95% CI	
		Satisfied	Dissatisfied	Corollary (95% CI)	AOR (95% CI)
Age (years)	18–39	218 (70.1)	93 (29.9)	1^a^	1
	40–55	60 (82.2)	13 (17.8)	1.97 (1.03, 3.76)^*∗*^	2.0 (0.79, 5.19)
	>55	24 (70.6)	10 (29.4)	1.02 (0.47, 2.22)	0.68 (0.22, 2.03)
Body mass index	Normal weight	236 (72.6)	89 (27.4)	1^a^	1
	Over weight	44 (83)	9 (17)	1.84 (0.86, 3.930	1.47 (0.56, 3.87)
	Obese	6 (54.5)	5 (45.5)	0.45 (0.14, 1.52)	0.73 (0.13, 4.15)
	Under weight	16 (55.2)	13 (44.8)	0.46 (0.22, 1.00)	0.59 (0.17, 1.44
ASA	ASA1	222 (71.4)	89 (28.6)	1.81 (0.91, 3.61)	3.55 (1.20, 10.55)^*∗*^
	ASA2	58 (84.1)	11 (15.9)	3.84 (1.54, 9.54)^1^	3.72 (1.04, 13.28)^*∗*^
	ASA3 and ASA4	22 (57.9)	16 (42.1)	1^a^	1^b^
Body site of surgery	Limbs	32 (68.1)	15 (31.9)	1^a^	1
	Head and neck	39 (75)	13 (25)	1.41 (0.59, 3.38)	1.06 (0.28, 3.84)
	Thoracic	7 (87.5)	1 (12.5)	3.28 (0.37, 29.12)	3.24 (0.23, 46.39)
	Abdominal	189 (70.3)	80 (29.7)	1.12 (0.57, 2.16)	0.66 (0.24, 1.78)
	Spine	4 (100)	0 (0)		
	Genitourinary	31 (81.6)	7 (18.4)	2.08 (0.75, 5.78)	3.37 (0.68, 16.66)
Type of surgery	Elective	159 (84.6)	29 (15.4)	3.34 (2.07, 5.38)^*∗∗*^	0.41 (0.15, 1.10)
	Emergency	143 (62.2)	87 (37.8)	1^a^	1
PONB	Yes	154 (95.7)	7 (4.3)	16.20 (7.30, 35.95)^*∗∗*^	9.14 (3.93, 20.86)^*∗∗*^
	No	148 (57.6)	109 (42.4)	1^a^	1^b^
Analgesics	Yes	297 (73.7)	106 (26.3)	5.6 (1.87, 16.77)^*∗*^	6.10 (1.17, 33.91)^*∗*^
	No	5 (33.3)	10 (66.7)	1^a^	1^b^
Information of POPM	Yes	38 (61.3)	24 (38.7)	0.55 (0.31, 0.97)^*∗*^	0.49 (0.22, 1.08)
	No	264 (74.2)	92 (25.8)	1^a^	1
Intraoperative pain	Yes	42 (58.3)	30 (41.7)	1^a^	1
	No	260 (75.1)	86 (24.9)	2.16 (1.27, 3.66)^*∗*^	1.31 (0.61, 2.85)
Postoperative pain	Yes	80 (57.3)	69 (46.3)	1^a^	1^b^
	No	222 (82.5)	47 (17.5)	4.07 (2.6, 6.48)^*∗∗*^	1.86 (1.02, 3.39)^*∗*^
Analgesic before request	Yes	260 (86.4)	41 (13.6)	11.32 (6.86, 18.69)^*∗∗*^	6.90 (3.72, 12.83)^*∗∗*^
	No	42 (35.9)	75 (64.1)	1^a^	1^b^
Analgesic when need it	Yes	56 (72.7)	21 (27.3)	1.03 (0.59, 1.79)	
	No	246 (72.1)	95 (27.9)	1	

^*∗*^= *P* value < 0.05, ^*∗∗*^=*P* < 0.001, ^a^ = significant from bivariate logistic regression model, and ^b^ = significant from multivariate logistic regression model. PRNB: peripheral nerve block. POPM: postoperative pain management.

## Data Availability

All data generated or analyzed during this study are included in this published article.

## Abbreviations

ASA: American Society of Anesthesiologists
SPSS: Statistical Package for Social Sciences
BMI: Body mass index
SD: Standard deviation
GA: General anesthesia
CI: Confidence interval
COR: Crude odds ratio
AOR: Adjusted odds ratio
POPM: Postoperative pain management
PONB: Postoperative nerve block.
